# Association between plasma total homocysteine level within normal range and bone mineral density in adults

**DOI:** 10.1186/s13018-020-02012-x

**Published:** 2020-10-15

**Authors:** Zhongxin Zhu, Changhua Liu, Xiao’e Li, Xiaocong Yao

**Affiliations:** 1 Department of Osteoporosis Care and Control, Xiaoshan First Affiliated Hospital of Hangzhou Normal University, No.199, Shixin South Road, Xiaoshan District, Hangzhou, 311200 Zhejiang Province People’s Republic of China; 2 Department of Hematology, Xiaoshan First Affiliated Hospital of Hangzhou Normal University, Hangzhou, 311200 Zhejiang China

**Keywords:** Homocysteine, Bone health, Biomarker, NHANES, Cross-sectional study

## Abstract

**Background:**

Growing evidence indicates that homocysteine is a noteworthy marker for general health status. However, research regarding plasma total homocysteine (tHcy) levels and bone mineral density (BMD) is sparse and controversial. Hence, we aimed to investigate the association between plasma tHcy level within normal range and lumbar BMD in adults.

**Methods:**

In this cross-sectional study, using the National Health and Nutrition Examination Survey database, data on 10748 adults aged between 30 and 85 years were analyzed. The weighted multiple logistic regression analyses were conducted to evaluate the association between plasma tHcy level and lumbar BMD. The fitted smoothing curves were performed to explore potential non-linear relationships. When non-linearity was detected, we further calculated the inflection point using a recursive algorithm and constructed a weighted two-piecewise linear regression model.

**Results:**

After adjusting for all the covariates, the association between plasma tHcy and lumbar BMD was different in various age groups (adults aged 30–49 years: *β* = −0.0004, 95% CI −0.0025, 0.0018; adults aged 50–69 years: *β* = 0.0001, 95% CI −0.0025, 0.0026; adults aged 70–85 years: *β* = 0.0050, 95% CI 0.0008, 0.0092). In the subgroup analysis stratified by gender, this association also differed based on gender. There was a negative trend in females (aged 30–49 years: *β* = −0.0022, 95% CI −0.0054, 0.0011; aged 50–69 years: *β* = −0.0028, 95% CI −0.0062, 0.0007), and a positive trend in males (aged 30–49 years: *β* = 0.0018, 95% CI −0.0012, 0.0048; aged 50–69 years: *β* = 0.0027, 95% CI −0.0009, 0.0063) in both 30–49 years group and 50–69 years group. In the 70–85 years group, this association was significantly positive in males (*β* = 0.0136, 95% CI 0.0068, 0.0204), but was not significantly different in females (*β* = 0.0007, 95% CI −0.0046, 0.0060).

**Conclusion:**

The correlation between plasma tHcy level within the normal range and lumbar BMD differs by age and gender.

## Introduction

Homocysteine (Hcy) is a non-essential amino acid derived from methionine metabolism. Growing evidence indicates that Hcy is a noteworthy marker for general health status, and increased plasma total homocysteine (tHcy) level is regarded as an independent risk factor for various human diseases, including cardiovascular diseases, chronic kidney diseases, neurological disorders, and bone tissue damages [1].

In the clinical diagnosis of osteoporosis and prediction of the risk of osteoporotic fracture, bone mineral density (BMD) is a commonly used quantitative indicator [2]. Nevertheless, bone turnover markers (BTMs) are preferable as dynamic indices for evaluating the status of bone turnover and progression after drug administration during the clinical treatment of osteoporosis. Plasma tHcy level, as a bone matrix-related marker, has been mentioned in the clinical guideline for the diagnosis and treatment of osteoporosis [3]. However, the mechanisms underlying the relationship between plasma tHcy level and BMD have not yet been unraveled. Besides, data regarding plasma tHcy level and BMD are relatively sparse, and inconsistent results regarding their relationship, including inverse [4–11], mixed [12–14], and no associations [15–21], have been reported. Therefore, we used the National Health and Nutrition Examination Survey (NHANES) database to investigate the relationship of plasma tHcy level within the normal range with lumbar BMD in adults aged between 30 and 85 years.

## Methods

### Study participants

The data of this study were obtained from the NHANES (1999–2006), which was an ongoing survey conducted by the Center for Disease Control and Prevention (CDC). CDC used a multistage, complex clustered probability design to select a representative sample of non-institutionalized United States civilians. The survey data are made available on the internet for researchers. A total of 11901 participants aged between 30 and 85 years remained after the exclusion of 1903 subjects with missing plasma tHcy data, 1405 subjects with missing lumbar BMD data, and 1236 subjects with cancer. We further excluded 1153 subjects with plasma tHcy levels outside the normal physiological range of 5–15 μmol/L [22], resulting in a final study population of 10748 people. All protocols were approved by the research ethical review board of the National Center for Health Statistics (NCHS), and informed consent forms were obtained from all participants.

### Study variables

The Abbott Imx was used to determine plasma tHcy level for NHANES 1999–2001, and the Abbott AxSym was used starting NHANES 2002. The certified radiology technologists used standard radiologic techniques and protocols to measure and analyze lumbar BMD on a Hologic QDR-4500A fan-beam densitometer. Age, gender, race, income-poverty ratio, education level, alcohol consumption, smoking behavior, physical activity, body mass index, blood urea nitrogen, total protein, total cholesterol, serum uric acid, serum folate, serum vitamin B_12_, serum phosphorus, and serum calcium were considered potential covariates and confounding factors, which were adjusted in the analytic models. The detailed information on the variables in this study can be found at www.cdc.gov/nchs/nhanes/.

### Statistical analysis

The NHANES sample weights were used as recommended by the NCHS. Statistical analyses were performed using R (version 3.5.3) and the EmpowerStats software (http://www.empowerstats.com). *P* values <0.05 were considered statistically significant. The weighted linear regression models were used to analyze the difference between dichotomous variables, and the weighted chi-square tests were used for continuous variables. The weighted multivariate linear regression analyses were conducted for examining the association between plasma tHcy level and lumbar BMD. The smooth curve fittings were further performed to explore their potential non-linear relationships. When non-linearity was detected, we further calculated the inflection point using a recursive algorithm and constructed a weighted two-piecewise linear regression model.

## Results

Table 1 shows the basic characteristics of 10748 participants included in the present study. Compared with the plasma tHcy quartile 1 group, the other quartile groups were older, more likely to be males, had higher blood urea nitrogen, total protein, total cholesterol, serum uric acid, serum phosphorus, and serum calcium, and had lower serum folate and vitamin B_12_.

**Table 1:** Description of 10748 participants included in the present study

Plasma total homocysteine quartiles (μmol/L)	Q1 (5-6.86)	Q2 (6.87-8.22)	Q3 (8.23-9.95)	Q4 (9.96-15)	P value
Age (years)	44.31 ± 10.71	47.24 ± 11.86	50.15 ± 12.76	55.27 ± 14.31	<0.001
Gender (%)					<0.001
Male	31.07	48.73	61.36	65.57	
Female	68.93	51.27	38.64	34.43	
Race (%)					<0.001
Non-Hispanic White	66.75	73.54	75.27	76.86	
Non-Hispanic Black	10.76	10.33	10.58	10.82	
Mexican American	9.35	6.82	5.02	3.95	
Other race	13.13	9.31	9.13	8.37	
Body mass index (kg/m2)	28.31 ± 6.70	28.86 ± 6.49	28.60 ± 5.90	28.81 ± 6.03	0.004
Education level (%)					<0.001
Less than high school	18.49	17.68	18.73	22.24	
High school	22.56	25.37	26.40	28.40	
More than high school	58.95	56.96	54.88	49.36	
Income-poverty ratio	3.19 ± 1.56	3.27 ± 1.52	3.26 ± 1.54	3.04 ± 1.53	<0.001
Physical activity (%)					<0.001
Not walk very much	14.66	15.03	15.84	19.79	
Walk a lot	27.89	27.38	26.34	27.87	
Climb often	21.30	20.47	17.30	15.48	
Heavy activity	30.60	31.00	32.64	28.28	
Not recorded	5.54	6.13	7.88	8.58	
Smoking behavior (%)					<0.001
None	57.23	51.12	46.64	41.37	
Past	22.45	26.51	28.00	29.13	
Current	20.32	22.36	25.35	29.50	
Alcohol consumption (%)					<0.001
Non-drinker	33.22	32.40	29.99	36.10	
Moderate alcohol use	41.36	35.15	33.15	30.16	
High alcohol use	25.42	32.45	36.86	33.74	
Blood urea nitrogen (mg/dL)	12.39 ± 3.81	12.99 ± 3.92	13.69 ± 4.32	14.57 ± 5.65	<0.001
Total protein (mg/dL)	7.25 ± 0.46	7.26 ± 0.46	7.29 ± 0.46	7.31 ± 0.50	<0.001
Total cholesterol (mg/dL)	201.1 ± 37.3	205.8 ± 40.2	210.1 ± 42.1	210.4 ± 45.9	<0.001
Serum uric acid (mg/dL)	4.81 ± 1.20	5.30 ± 1.30	5.59 ± 1.32	6.01 ± 1.39	<0.001
Serum folate (ng/mL)	15.75 ± 10.20	14.82 ± 15.76	14.07 ± 11.01	13.04 ± 9.07	<0.001
Serum vitamin B_12_( pg/mL)	611.6 ± 1758.3	534.8 ± 314.4	490.0 ± 240.1	450.8 ± 299.7	<0.001
Serum phosphorus (mg/dL)	3.63 ± 0.53	3.67 ± 0.56	3.68 ± 0.56	3.68 ± 0.57	0.002
Serum calcium (mg/dL)	9.39 ± 0.35	9.46 ± 0.36	9.52 ± 0.35	9.57 ± 0.38	<0.001
Lumbar bone mineral density (g/cm2)	1.05 ± 0.15	1.04 ± 0.15	1.04 ± 0.16	1.04 ± 0.17	0.054

Mean ± SD for continuous variables: *P* value was calculated by the weighted linear regression model.

% for categorical variables: *P* value was calculated by the weighted chi-square test

The results of different multivariate linear regression models for adults aged between 30–49 years, 50–69 years, and 70–85 years are shown in Tables 2, 3, and 4, respectively. After adjusting for all the covariates presented in Table 1, the association between plasma tHcy level and lumbar BMD differed by age (adults aged 30–49 years: *β* = −0.0004, 95% CI −0.0025, 0.0018; adults aged 50–69 years: *β* = 0.0001, 95% CI −0.0025, 0.0026; adults aged 70–85 years: *β* = 0.0050, 95% CI 0.0008, 0.0092). In the subgroup analysis stratified by gender, this association also differed based on gender. There was a negative trend in females (aged 30–49 years: *β* = −0.0022, 95% CI −0.0054, 0.0011; aged 50–69 years: *β* = −0.0028, 95% CI −0.0062, 0.0007), and a positive trend in males (aged 30–49 years: *β* = 0.0018, 95% CI −0.0012, 0.0048; aged 50–69 years: *β* = 0.0027, 95% CI −0.0009, 0.0063) in both 30–49 years group and 50–69 years group. In the 70–85 years group, this association was significantly positive in males (*β* = 0.0136, 95% CI 0.0068, 0.0204), but was not significantly different in females (*β* = 0.0007, 95% CI −0.0046, 0.0060). In the subgroup analysis stratified by race, this association again differed by age and gender (Tables 2, 3, and 4).

**Table 2:** Association of plasma total homocysteine (umol/L) with lumbar bone mineral density (g/cm2) in adults aged 30–49 years (*n* = 4977)

	Model 1*β* (95% CI)	Model 2*β* (95% CI)	Model 3*β* (95% CI)
Plasma total homocysteine	−0.0025 (−0.0046, -0.0005)	−0.0012 (−0.0033, 0.0009)	−0.0004 (−0.0025, 0.0018)
Stratified by gender			
Male	0.0007 (−0.0022, 0.0036)	−0.0001 (−0.0030, 0.0028)	0.0018 (−0.0012, 0.0048)
Female	-0.0025 (-0.0057, 0.0007)	−0.0025 (−0.0057, 0.0006)	−0.0022 (−0.0054, 0.0011)
Stratified by race			
Non-Hispanic White	−0.0045 (−0.0074–0.0016)	−0.0023 (−0.0054, 0.0008)	−0.0007 (−0.0040, 0.0026)
Non-Hispanic Black	0.0018 (−0.0028, 0.0064)	0.0038 (−0.0012, 0.0087)	0.0047 (−0.0005, 0.0100)
Mexican American	-0.0014 (-0.0059, 0.0030)	0.0028 (−0.0019, 0.0076)	0.0015 (−0.0034, 0.0064)
Other race/ethnicity	-0.0040 (-0.0107, 0.0027)	−0.0032 (−0.0106, 0.0041)	−0.0009 (−0.0088, 0.0071)

Model 1: no covariates were adjusted.

Model 2: age, gender, race were adjusted.

Model 3: age, gender, race, body mass index, education level, income-poverty ratio, physical activity, smoking behavior, alcohol consumption, blood urea nitrogen, total protein, total cholesterol, serum uric acid, serum folate, serum vitamin B_12_, serum phosphorus, and serum calcium were adjusted.

In the subgroup analysis stratified by gender or race, the model is not adjusted for the stratification variable itself

**Table 3:** Association of plasma total homocysteine (umol/L) with lumbar bone mineral density (g/cm2) in adults aged 50–69 years (*n* = 4123)

	Model 1β (95% CI)	Model 2β (95% CI)	Model 3β (95% CI)
Plasma total homocysteine	0.0040 (0.0016, 0.0063)	0.0002 (−0.0022, 0.0026)	0.0001 (−0.0025, 0.0026)
Stratified by gender			
Male	0.0048 (0.0013, 0.0082)	0.0025 (−0.0009, 0.0059)	0.0027 (−0.0009, 0.0063)
Female	−0.0026 (−0.0059, 0.0007)	−0.0017 (−0.0050, 0.0016)	−0.0028 (−0.0062, 0.0007)
Stratified by race			
Non-Hispanic White	0.0025 (−0.0008, 0.0058)	−0.0002 (−0.0036, 0.0032)	−0.0003 (−0.0040, 0.0034)
Non-Hispanic Black	0.0040 (−0.0013, 0.0093)	0.0005 (−0.0049, 0.0059)	0.0007 (−0.0052, 0.0066)
Mexican American	0.0050 (−0.0001, 0.0101)	0.0008 (−0.0045, 0.0062)	0.0018 (−0.0038, 0.0075)
Other race/ethnicity	0.0032 (−0.0050, 0.0115)	0.0028 (−0.0061, 0.0116)	0.0021 (−0.0073, 0.0115)

Model 1: no covariates were adjusted.

Model 2: age, gender, race/ethnicity were adjusted.

Model 3: age, gender, race, body mass index, education level, income-poverty ratio, physical activity, smoking behavior, alcohol consumption, blood urea nitrogen, total protein, total cholesterol, serum uric acid, serum folate, serum vitamin B_12_, serum phosphorus, and serum calcium were adjusted.

In the subgroup analysis stratified by gender or race, the model is not adjusted for the stratification variable itself

**Table 4:** Association of plasma total homocysteine (umol/L) with lumbar bone mineral density (g/cm2) in adults aged 70-85 years (n = 1648).

	Model 1β (95% CI)	Model 2β (95% CI)	Model 3β (95% CI)
Plasma total homocysteine	0.0103 (0.0061, 0.0144)	0.0053 (0.0013, 0.0093)	0.0050 (0.0008, 0.0092)
Stratified by gender			
Male	0.0107 (0.0043, 0.0170)	0.0096 (0.0031, 0.0162)	0.0136 (0.0068, 0.0204)
Female	0.0015 (-0.0035, 0.0065)	0.0024 (-0.0027, 0.0075)	0.0007 (-0.0046, 0.0060)
Stratified by race			
Non-Hispanic White	0.0097 (0.0046, 0.0149)	0.0050 (-0.0001, 0.0100)	0.0047 (-0.0006, 0.0100)
Non-Hispanic Black	0.0126 (0.0001, 0.0251)	0.0179 (0.0050, 0.0308)	0.0139 (0.0004, 0.0273)
Mexican American	0.0002 (-0.0160, 0.0163)	-0.0040 (-0.0228, 0.0149)	0.0040 (-0.0190, 0.0270)
Other race/ethnicity	0.0095 (0.0054, 0.0136)	0.0053 (0.0013, 0.0093)	0.0050 (0.0008, 0.0092)

Model 1: no covariates were adjusted.

Model 2: age, gender, race/ethnicity were adjusted.

Model 3: age, gender, race, body mass index, education level, income-poverty ratio, physical activity, smoking behavior, alcohol consumption, blood urea nitrogen, total protein, total cholesterol, serum uric acid, serum folate, serum vitamin B_12_, serum phosphorus, and serum calcium were adjusted.

In the subgroup analysis stratified by gender or race, the model is not adjusted for the stratification variable itself.

**Fig. 1:**
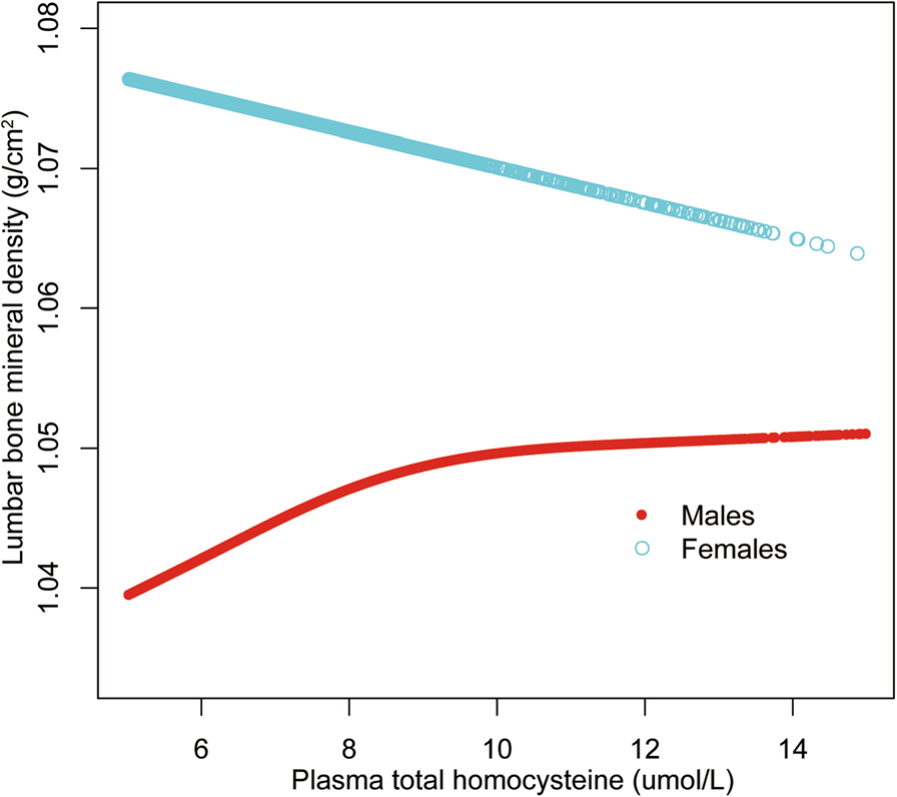
The association between plasma total homocysteine and lumbar bone mineral density in adults aged 30–49 years, stratified by gender. Age, race, body mass index, education level, income-poverty ratio, physical activity, smoking behavior, alcohol consumption, blood urea nitrogen, total protein, total cholesterol, serum uric acid, serum folate, serum vitamin B12, serum phosphorus, and serum calcium were adjusted

**Fig. 2.**
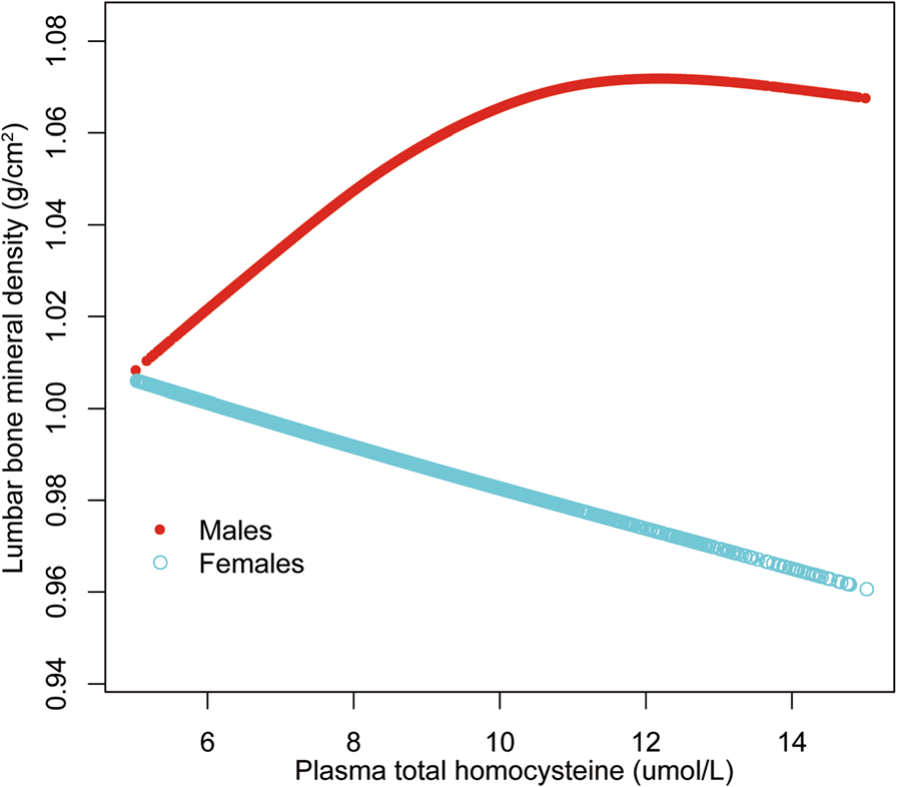
The association between plasma total homocysteine and lumbar bone mineral density in adults aged 50–69 years, stratified by gender. Age, race, body mass index, education level, income-poverty ratio, physical activity, smoking behavior, alcohol consumption, blood urea nitrogen, total protein, total cholesterol, serum uric acid, serum folate, serum vitamin B12, serum phosphorus, and serum calcium were adjusted

**Fig. 3:**
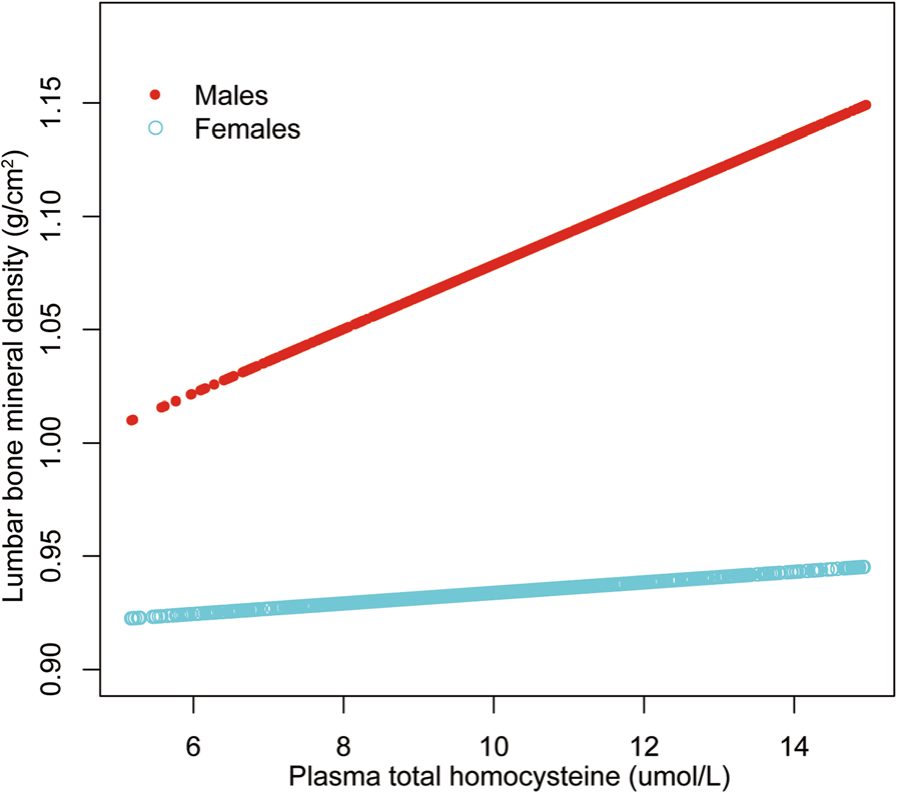
The association between plasma total homocysteine and lumbar bone mineral density in adults aged 70–85 years, stratified by gender. Age, race, body mass index, education level, income-poverty ratio, physical activity, smoking behavior, alcohol consumption, blood urea nitrogen, total protein, total cholesterol, serum uric acid, serum folate, serum vitamin B12, serum phosphorus, and serum calcium were adjusted

We further explored the potential non-linear relationship of plasma tHcy level and lumbar BMD using smooth curve fittings (Figs. 1, 2 and 3). Non-linear relationships of plasma tHcy levels with lumbar BMD in males aged 30–49 years and 50–69 years were detected. We further calculated the inflection points at 9.0 μmol/L for males aged 30–49 years and 10.0 μmol/L for 50–69 years (Table 5).

**Table 5:** Threshold effect analysis of plasma total homocysteine on lumbar bone mineral density using a two-piecewise linear regression model

Lumbar bone mineral density	Adjusted ß (95% CI)
Plasma total homocysteine	
Males aged 30-49 years	
Fitting by standard linear model	0.0018 −0.0012, 0.0048)
Fitting by two-piecewise linear model	
Inflection point	9.0
Plasma total homocysteine <9.0 (umol/L)	0.0042 (−0.0015, 0.0099)
Plasma total homocysteine >9.0 (umol/L)	-0.0005 (−.0059, 0.0050)
Log-likelihood ratio	0.323
Males aged 50-69 years	
Fitting by standard linear model	0.0027 (−0.0009, 0.0063)
Fitting by two-piecewise linear model	
Inflection point	10.0
Plasma total homocysteine <10.0 (umol/L)	0.0098 (0.0032, 0.0163)
Plasma total homocysteine >10.0 (umol/L)	−0.0053 −0.0125, 0.0019)
Log-likelihood ratio	0.011

Age, race, body mass index, education level, income-poverty ratio, physical activity, smoking behavior, alcohol consumption, blood urea nitrogen, total protein, total cholesterol, serum uric acid, serum folate, serum vitamin B_12_, serum phosphorus, and serum calcium were adjusted

## Discussion

In this population-based study of US adults, we found that (1) the association between plasma tHcy level and lumbar BMD was different in various age groups; (2) this association was also different by gender. To the best of our knowledge, this study is thus far the largest sample size study on the relationship between plasma tHcy level and BMD in a general adult population.

It was reported that Hcy could affect proper bone metabolism. In vitro, high Hcy levels can modulate the bone remodeling process by inhibiting osteoblastic differentiation; inducing apoptosis in osteoblasts, osteocytes, and bone marrow stromal cells; and increasing osteoclast activity [23]. In an experimental rat model with hyperhomocysteinemia induced by a 2.4% methionine-enriched diet for 12 weeks, there was a significant decrease in the bone formation marker osteocalcin and an increase in urinary N-terminal collagen I telopeptides compared with normal rats [24]. In the same rat model with hyperhomocysteinemia, a 19% reduction occurred in the bone volume at the femoral neck and a 45% reduction at the distal femur [25].

The relationship of the Hcy level with BMD has been studied in various populations, but the conclusions remain inconsistent. Some studies demonstrated that high Hcy levels were associated with lower BMD [4–11], whereas other studies found no significant correlation between them [15–21]. In particular, unlike all the mentioned research, some studies reported mixed correlations. The results of a cross-sectional survey of 446 post-menopausal women showed that tHcy levels were negatively associated with BMD of the total femur, but not of the femoral neck or lumbar spine [13]. The results of a cross-sectional study of 3337 healthy Korean adults suggested that the correlation of Hcy with BMD was different based on sex and age, and BMD of the lumbar spine and femur decreased as the tHcy levels increased in women aged <50 years, but no significant correlation was found in all age groups of men or other age groups of women [14]. The results of the Hordaland Homocysteine Study (3070 women and 2268 men, aged 47–50 and 71–75 years) suggest that plasma tHcy level is an independent risk factor for low BMD in women but not in men [12]. In our study, we conducted subgroup analyses to make a better use of the data following the STrengthening the Reporting of OBservational studies in Epidemiology (STROBE) guideline [26]. Further, we found that the association between plasma tHcy level and lumbar BMD differed by age and gender. Therefore, these conflicting conclusions may be attributed to the heterogeneity among studies, including study design, study size, and differences in participants’ selection, such as age and gender.

The size is the major strength of this study; we investigated plasma tHcy and lumbar BMD of 10748 samples of the multiracial population. Thus, subgroup analyses could be performed due to the large sample size. In addition, we used smooth curve fittings to explore potential non-linear relationships. However, some limitations should be noted. The cross-sectional nature of the study precludes any inferences about causality. Besides, because the participants with cancer were excluded, study results cannot be generalized to these special populations. Furthermore, the data of this study were analyzed with no exclusion of other diseases that may influence bone health. This makes the results more generalizable but may weaken the observed association.

In conclusion, we found that the correlation between plasma tHcy level within normal range and lumbar BMD differed according to age and gender. Our findings provide new insights to advance the research of the link between Hcy and bone health.

## Data Availability

The survey data are publicly available on the internet for data users and researchers throughout the world.

## Abbreviations

Hcy: Homocysteine
tHcy: Total homocysteine
BMD: Bone mineral density
BTMs: Bone turnover markers
NHANES: National Health and Nutrition Examination Survey
CDC: The Center for Disease Control and Prevention
NCHS: The National Center for Health Statistics
STROBE: STrengthening the Reporting of OBservational studies in Epidemiology

## References

[1] Skovierova H, Vidomanova E, Mahmood S, Sopkova J, Drgova A, Cervenova T, Halasova E, Lehotsky J. The molecular and cellular effect of homocysteine metabolism imbalance on human health. Int J Mol Sci. 2016;17(10). 10.3390/ijms17101733 PMC5085763 27775595

[2] Zhu Y, Shen J, Cheng Q, Fan Y, Lin W (2016). Plasma homocysteine level is a risk factor for osteoporotic fractures in elderly patients. Clin Interventions Aging.

[3] Nishizawa Y, Miura M, Ichimura S, Inaba M, Imanishi Y, Shiraki M, Takada J, Chaki O, Hagino H, Fukunaga M (2019). Executive summary of the Japan Osteoporosis Society Guide for the use of bone turnover markers in the diagnosis and treatment of osteoporosis (2018 Edition). Clinica Chimica Acta.

[4] Gerdhem P, Ivaska KK, Isaksson A, Pettersson K, Vaananen HK, Obrant KJ, Akesson K (2007). Associations between homocysteine, bone turnover, BMD, mortality, and fracture risk in elderly women. J Bone Mineral Res.

[5] Enneman AW, Swart KM, Zillikens MC, van Dijk SC, van Wijngaarden JP, Brouwer-Brolsma EM, Dhonukshe-Rutten RA, Hofman A, Rivadeneira F, van der Cammen TJ (2014). The association between plasma homocysteine levels and bone quality and bone mineral density parameters in older persons. Bone.

[6] Alvarez-Cienfuegos A, Cantero-Nieto L, Garcia-Gomez JA, Callejas-Rubio JL, Gonzalez-Gay MA, Ortego-Centeno N. Association between homocysteine serum level and bone mineral density in patients with rheumatoid arthritis. J Clin Densitometry. 2019. 10.1016/j.jocd.2019.03.008 31005409

[7] Saoji R, Das RS, Desai M, Pasi A, Sachdeva G, Das TK, Khatkhatay MI (2018). Association of high-density lipoprotein, triglycerides, and homocysteine with bone mineral density in young Indian tribal women. Archives of osteoporosis.

[8] Bahtiri E, Islami H, Rexhepi S, Qorraj-Bytyqi H, Thaci K, Thaci S, Karakulak C, Hoxha R (2015). Relationship of homocysteine levels with lumbar spine and femur neck BMD in postmenopausal women. Acta Reumatologica Portuguesa.

[9] Ebesunun MO, Umahoin KO, Alonge TA, Adebusoye LA (2014). Plasma homocysteine, B vitamins and bone mineral density in osteoporosis: a possible risk for bone fracture. Afr J Med Med Sci.

[10] Rehackova P, Skalova S, Kutilek S (2013). Serum homocysteine levels in children and adolescents with impaired bone health. Revista Brasileira de Reumatologia.

[11] Ouzzif Z, Oumghar K, Sbai K, Mounach A, el Derouiche M, El Maghraoui A (2012). Relation of plasma total homocysteine, folate and vitamin B12 levels to bone mineral density in Moroccan healthy postmenopausal women. Rheumatol Int.

[12] Gjesdal CG, Vollset SE, Ueland PM, Refsum H, Drevon CA, Gjessing HK, Tell GS (2006). Plasma total homocysteine level and bone mineral density: the Hordaland Homocysteine Study. Arch Internal Medicine.

[13] Bucciarelli P, Martini G, Martinelli I, Ceccarelli E, Gennari L, Bader R, Valenti R, Franci B, Nuti R, Mannucci PM (2010). The relationship between plasma homocysteine levels and bone mineral density in post-menopausal women. Eur J Internal Med.

[14] Kim JI, Moon JH, Chung HW, Kong MH, Kim HJ (2016). Association between homocysteine and bone mineral density according to age and sex in healthy adults. J Bone Metabolism.

[15] Perier MA, Gineyts E, Munoz F, Sornay-Rendu E, Delmas PD (2007). Homocysteine and fracture risk in postmenopausal women: the OFELY study. Osteoporosis Int.

[16] Cagnacci A, Baldassari F, Rivolta G, Arangino S, Volpe A (2003). Relation of homocysteine, folate, and vitamin B12 to bone mineral density of postmenopausal women. Bone.

[17] van Meurs JB, Dhonukshe-Rutten RA, Pluijm SM, van der Klift M, de Jonge R, Lindemans J, de Groot LC, Hofman A, Witteman JC, van Leeuwen JP (2004). Homocysteine levels and the risk of osteoporotic fracture. New Eng J Med.

[18] Rumbak I, Zizic V, Sokolic L, Cvijetic S, Kajfez R, Colic Baric I (2012). Bone mineral density is not associated with homocysteine level, folate and vitamin B12 status. Archiv Gynecol Obstet.

[19] Elshorbagy AK, Gjesdal CG, Nurk E, Tell GS, Ueland PM, Nygard O, Tverdal A, Vollset SE, Smith AD, Refsum H (2009). Cysteine, homocysteine and bone mineral density: a role for body composition?. Bone.

[20] Kaya B, Ates E, Paydas S, Sertdemir Y, Balal M (2019). Evaluation of the relationship between homocysteine, parathormone, vitamin d3, and bone mineral densitometry in recipients of kidney transplant. Transplant Proceed.

[21] Haroon NN, Marwaha RK, Godbole MM, Gupta SK (2012). Role of B(1)(2) and homocysteine status in determining BMD and bone turnover in young Indians. J Clin Densitometry.

[22] Buckner SL, Loenneke JP, Loprinzi PD (2017). Single and combined associations of accelerometer-assessed physical activity and muscle-strengthening activities on plasma homocysteine in a national sample. Clin Physiol Function Imaging.

[23] Saito M, Marumo K (2018). The effects of homocysteine on the skeleton. Curr Osteoporosis Rep.

[24] Ozdem S, Samanci S, Tasatargil A, Yildiz A, Sadan G, Donmez L, Herrmann M (2007). Experimental hyperhomocysteinemia disturbs bone metabolism in rats. Scand J Clin Lab Investigation.

[25] Herrmann M, Wildemann B, Claes L, Klohs S, Ohnmacht M, Taban-Shomal O, Hubner U, Pexa A, Umanskaya N, Herrmann W (2007). Experimental hyperhomocysteinemia reduces bone quality in rats. Clin Chem.

[26] von Elm E, Altman DG, Egger M, Pocock SJ, Gotzsche PC, Vandenbroucke JP (2007). The Strengthening the Reporting of Observational Studies in Epidemiology (STROBE) statement: guidelines for reporting observational studies. Lancet (London, England).

